# Two cases of serious rhabdomyolysis during linezolid treatment

**DOI:** 10.1007/s15010-016-0978-8

**Published:** 2017-01-12

**Authors:** Arno M. Lechner, Eva Past, Ulla Porsche, Jan M. Kern, Uta Hoppe, Ingrid Pretsch

**Affiliations:** 1 Division Medizinische Mikrobiologie, Paracelsus Medizinische Universität Salzburg, Universitätsinstitut für Medizinisch-Chemische Labordiagnostik, Muellner Hauptstrasse 48, 5020 Salzburg, Austria; 2 Landesapotheke Salzburg, Muellner Hauptstrasse 50, 5020 Salzburg, Austria; 3 Paracelsus Medizinische Universität Salzburg, Universitätsklinik für Innere Medizin II, Muellner Hauptstrasse 48, 5020 Salzburg, Austria

**Keywords:** Linezolid, Rhabdomyolysis, MRSA pneumonia, MRSA sepsis

## Abstract

Linezolid is an oxazolidinone antibiotic with activity against gram-positive organisms, particularly methicillin-resistant *Staphylococcus aureus* (MRSA). To the best of our knowledge, there are only two case reports on rhabdomyolysis in patients treated with linezolid. Here, we describe two cases of serious rhabdomyolysis: one in a patient with septic community-acquired (CA)-MRSA pneumonia and a second case in a patient with suspected catheter-related blood stream infection.

## Case 1

A 49-year-old Indian female living in Salzburg, Austria, had returned from a 3-week visit to India. Some days before hospitalization, she had noticed cough, fever up to 38.7 °C and worsening dyspnea. She was a type II diabetic patient treated with oral antidiabetics, but having lost her medicines during her travels, the antidiabetic treatment had been paused for 8 days prior to presentation. In the emergency department (ED), the patient was seriously dehydrated, somnolent and showed Kussmaul-respiration pattern. The laboratory tests revealed a blood glucose level of 469 mg/dL, C-reactive protein (CRP) of 34.0 mg/dL, lactate of 4.00 mmol/L, pH of 7.207 and a base excess of −19.2 mmol/L. Leukocytes, platelets, transaminases and kidney function tests were within normal limits. The patient was admitted to the intensive care unit where she showed signs of septic shock a few hours later. She was intubated, mechanical ventilation and catecholamines and intravenous (IV) insulin were started. An empiric broad spectrum antibiotic therapy with meropenem and azithromycin was administered instead of amoxicillin/clavulanic acid and clarithromycin previously given in the ED. On day 2, reconsidering the patient´s recent travel history and potential exposure to CA-MRSA [1], linezolid was added. On day 3, azithromycin was stopped and teicoplanin was started due to positive blood cultures for methicillin-resistant, Panton-Valentine leucocidin producing *Staphylococcus aureus* (PVL + MRSA). Teicoplanin was added to linezolid to compensate for the variability of linezolid plasma concentrations in critically ill patients [2] as was shown by the subtherapeutic plasma trough level of linezolid on day 4. To limit nephrotoxicity, teicoplanin was preferred to vancomycin. Bronchoalveolar lavage specimens from the same day also grew PVL + MRSA.

A chest X-ray revealed an extensive infiltrate of the right lung and satellite infiltrates on the left side. Influenza A/B RNA PCR, Influenza A H1N1/9 PCR, urine antigen tests for pneumococci and *Legionella* spp., a malaria blood smear and serological testing for dengue virus were all negative. Transthoracic and transesophageal echocardiography performed on admission and on day 10 did not detect any vegetations suspicious for infective endocarditis.

One day before starting IV linezolid 600 mg twice daily, the patient’s creatine kinase (CK) blood concentration was 55 U/L (reference range 26–140 U/L). The first measurement of the patient’s linezolid trough level was performed on day 4 after four doses of linezolid 600 mg and showed a result of 0.1 µg/mL. At our laboratory, the therapeutic range for linezolid trough levels at steady state is 3.0–9.0 µg/mL. Therefore, the linezolid dosage was increased to 600 mg three times daily. On day 8, 5 days after the start of linezolid and 3 days after the dose increase, the linezolid plasma level reached the therapeutic level of 4.10 µg/mL. On day 3, 1 day after putting the patient on linezolid, the CK concentration started to rise above the upper limit of normal from 643 U/L on day 6, reaching a maximum of 11,988 U/L on day 11, the 10th day of linezolid treatment. Linezolid was discontinued at that point due to the suspicion that it may have caused rhabdomyolysis. From the following day, the CK concentration started to decline, reaching a level of 3,570 U/L after 1 week and 92 U/L another 2 weeks later. The relative fraction of CK-MB from total CK was within normal limits during the first 2 weeks and the level of high-sensitive troponin T remained normal (<14 ng/L) until day 10 and increased to a maximum of 685 ng/L on day 14. The patient also developed acute kidney injury with a twofold increase in serum creatinine (1 mg/dL on day 5 and 2 mg/dL on day 11).

An extensive clinical, radiological and pharmacological workup on differential diagnoses for rhabdomyolysis excluded bacterial pyomyositis and other causes of muscle injury such as intramuscular injections, surgical interventions, empyema, trauma, malignant neuroleptic syndrome or malignant hyperthermia. In addition, clinical criteria for staphylococcal toxic shock syndrome, which may complicate CA-MRSA-sepsis and could result in rhabdomyolysis, were not fulfilled according to the case definition of the Centers for Disease Control [3]. The patient had neither ingested alcohol nor used illicit drugs such as amphetamines, barbiturate or heroin. Also, she was not on any statin or any selective serotonin reuptake inhibitor (SSRI) or antipsychotics such as olanzapine, which are notorious for rhabdomyolysis.

An off-label trial of ceftaroline 600 mg twice daily was started on day 13 because the clinical result of the previous antibiotic therapy was not satisfying: the patient’s body temperatures remained high with progressive radiological and respiratory deterioration. In addition to extensive alveolar consolidations, computer tomography (CT) scans of the lungs demonstrated necrotic and subsequently cystic damage of tissue predominantly in the upper and lower lobes of the right lung. During treatment with ceftaroline, the patient’s condition improved constantly. When the patient was put on continuous hemofiltration, the ceftaroline dose was reduced to 300 mg twice daily. In summary, the patient received ventilator therapy for three weeks and continuous renal replacement therapy for five weeks. Seven weeks after admission, the patient was discharged to rehabilitation care. The patient has been doing well so far.

## Case 2

A 65-year-old patient was admitted to hospital due to deteriorating dyspnea and general weakness following a suspected viral respiratory infection. His medical history included cardiac failure NYHA III that had developed during the previous months and 40 pack-years of smoking. One week after admission, a coronary angiography (CAG) through the left radial artery was performed, which showed a 3-vessel coronary artery disease without an option for angioplastic or surgical intervention considering the results of cardiac nuclear and magnetic resonance imaging, where large myocardial scarified areas were demonstrated. As a complication of catheterization of the left radial artery, a critical ischemia of the left hand end-phalanges I–V resulted. Muscle damage was excluded at this point with CK values within normal limits. As drug therapy with parenteral alprostadil and unfractioned heparin had failed, an arteriovenous reverting procedure of the left vena cephalica was performed three weeks after the CAG. The ischemia of the fingers of left hand improved. At this time, there were no signs or symptoms of larger muscle damage of the left forearm such as a compartment syndrome. Due to increasing CRP values five weeks after admission reaching a maximum of 13.2 mg/dL (reference value <0.6 mg/dL) oral amoxicillin/clavulanic acid was started empirically. Four days later, due to a further increase of CRP to 15.6 mg/dL linezolid was added to cover suspected methicillin-resistent coagulase-negative staphylococci. On day 3 of linezolid therapy, when the linezolid trough level was 6.5 µg/mL (therapeutic range 3.0–9.0 µg/mL), CK concentration started to rise from normal values to 4,405 U/L, reaching a maximum of 10,072 U/l on day 5 of linezolid therapy. On this day, the administration of linezolid was stopped as there was no other plausible pharmacological or traumatological explanation for a CK rise and suspected rhabdomyolysis than linezolid. The patient had been on simvastatin 40 mg once daily before but the drug had been withheld for unknown reasons for more than 10 days prior to linezolid therapy. The patient was not on any antipsychotic, SSRI or other drugs susceptible for CK rise and rhabdomyolysis. During the subsequent days, CK concentrations halved daily, reaching normal values 14 days later. In parallel to the CK and myoglobin rise, the patient’s renal function deteriorated and renal replacement therapy was performed for 2 weeks.

## Discussion

Linezolid is an important therapeutic option in MRSA infections and sometimes as an empiric therapy in suspicion of gram-positive organisms resistant to beta-lactam antibiotics. Linezolid appears to be a better choice than vancomycin for the treatment of MRSA ventilator-associated pneumonia, and has been considered as the drug of choice for MRSA community-acquired pneumonia [4]. This might be due to the toxin-suppressing effect of linezolid as a protein-synthesis inhibitor and its excellent lung tissue penetration [5]. Vancomycin was the standard therapy in most MRSA bacteremia treatment studies [6]; however, in another study, there was no difference in terms of clinical cure in comparison to linezolid [7].

**Fig. 1:**
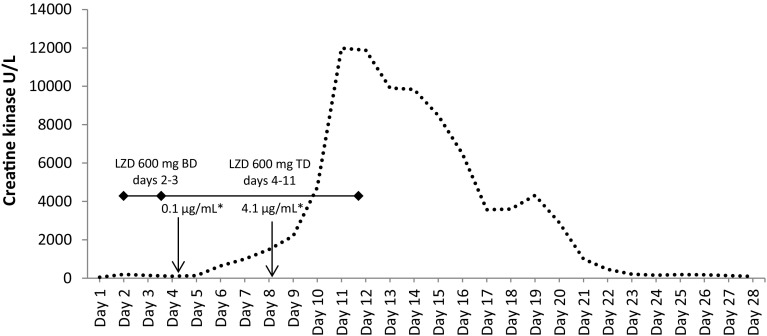
Case 1: rise in creatine kinase concentrations in parallel to linezolid treatment. *Asterisk* linezolid trough concentrations on day 4 and day 8 (reference range 3.0–9.0 µg/mL). *BD* denotes twice daily, *LZD* linezolid, *TD* three times daily

**Fig. 2:**
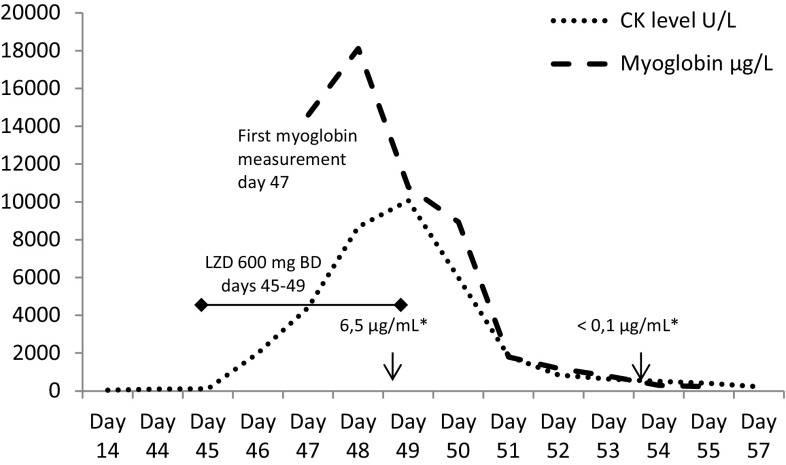
Case 2: rise in creatine kinase and myoglobin levels in parallel to linezolid treatment. *Asterisk* linezolid trough concentrations on day 49 and day 54 (reference range 3.0–9.0 µg/mL). *BD* denotes twice daily, *CK* creatine kinase, *LZD* linezolid

The most frequent side effects of linezolid are headache, diarrhea and nausea. Less frequent but more serious are hematological toxicities such as thrombocytopenia [8]. To the best of our knowledge, there are only two case reports on rhabdomyolysis in patients treated with linezolid in the literature: one in a patient with MRSA pneumonia [9] and the other in a patient treated for extensively drug-resistant tuberculosis [10]. In the latter, the authors provided evidence, that rhabdomyolysis was due to linezolid-induced inhibition of mitochondrial protein synthesis [10]. The myotoxic effect of linezolid in our first patient was potentially enhanced due to the exposure of increased doses of linezolid which had been necessary to reach the therapeutic range of the antibiotic. Of note, the plasma levels of linezolid in this case on day 4 and day 8 were below, respectively, within the therapeutic range, proving that the assumed myotoxic effect was not due to toxically high levels of the antibiotic. The timeline of development of rhabdomyolysis and its resolution in both cases corresponded strongly to the linezolid use suggesting a causal relationship of rhabdomyolysis and linezolid treatment (see Figs. 1, 2). The adverse drug reaction in both patients had a score of 8 on the drug reaction probability scale by Naranjo et al. [11]. This makes linezolid a probable agent for both adverse drug events, yet it is not definite. The start and stop of linezolid treatment corresponded exactly with increase, decrease and normalization of CK levels with a 2-day delay, whereas most of the co-administered medication in our first case during that time frame was continued or stopped much earlier than the CK level reached its maximum. The differentiation of CK to its isoenzyme CK-BB was not performed. Cerebral origin of the CK elevation was excluded clinically and by imaging studies such as CT- and MR-imaging scans of the brain. There was no other evidence of muscle tissue damage in both patients that could be detected by imaging procedures. Neither patient had been on any other drugs that are known to cause CK elevations. Rather, an idiosyncratic effect can be proposed in both patients, in particular in the first patient, as rhabdomyolysis developed despite the subtherapeutic level, when linezolid was commenced, but this remains speculative. To the best of our knowledge, rises of CK have not been described in initial clinical studies upon linezolid treatment and only two cases of rhabdomyolysis due to linezolid have been published. As these two additional cases occured in our institution within the relatively short period of 1 year, we speculate that some, perhaps milder forms of rhabdomyolysis may be overlooked in clinical practice.

## References

[1] Fomda BA, Thokar MA, Khan A, Bhat JA, Zahoor D, Bashir G, Majid A, Ray P (2014). Nasal carriage of methicillin-resistant *Staphylococcus aureus* among healthy population of Kashmir, India. Indian J Med Microbiol.

[2] Sazdanovic P, Jankovic SM, Kostic M, Dimitijevic A, Stefanovic S (2016). Pharmacokinetics of linezolid in critically ill patients. Expert Opin Drug Metab Toxicol.

[3] Toxic Shock Syndrome (other than streptococcal) (TSS). 2011 Case Definition [Internet]. Access via www.n.cdc.gov/nndss/conditions/toxic-shock-syndrome-other-than-streptococcal/case-definition/2011/on December 14, 2016.

[4] Wunderink RG (2013). How important is methicillin-resistant *Staphylococcus aureus* as a cause of community-acquired pneumonia and what is best antimicrobial therapy?. Infect Dis Clin North Am.

[5] Boselli E, Breilh D, Rimmelé T, Djabarouti S, Toutain J, Chassard D, Saux MC, Allaouchiche B (2005). Pharmacokinetics and intrapulmonary concentrations of linezolid administered to critically ill patients with ventilator-associated pneumonia. Crit Care Med.

[6] Holland TL, Arnold C, Fowler VG (2014). Clinical management of *Staphylococcus aureus* bacteremia: a review. JAMA.

[7] Shorr AF, Kunkel MJ, Kollef M (2005). Linezolid versus vancomycin for *Staphylococcus aureus* bacteraemia. J Antimicrob Chemother.

[8] Summary of product characteristics. Zyvoxid^®^ (Linezolid) 600 mg film-coated tablets. Accessed via https://aspregister.basg.gv.at on March 8, 2016.

[9] Allison GW, Perla RJ, Belliveau PP, Angelis SM (2009). Elevated creatine phosphokinase levels associated with linezolid therapy. Am J Health Syst Pharm.

[10] Carroll MW, Choi H, Min S, Hwang S, Park H, Song T, Park Y, Jeon HS, Goldfeder LC, Via LE, Lebron J, Jin B, Cai Y, Barry CE, Lee M (2012). Rhabdomyolysis in a patient treated with linezolid for extensively drug-resistant tuberculosis. Clin Infect Dis.

[11] Naranjo CA, Busto U, Sellers EM (1981). A method for estimating the probability of adverse drug reactions. Clin Pharmacol Ther.

